# Access to Health Care and Religion among Young American Men

**DOI:** 10.3390/ijerph6123225

**Published:** 2009-12-18

**Authors:** R. Frank Gillum, Nicole Jarrett, Thomas O. Obisesan

**Affiliations:** 1 College of Medicine, Howard University, Washington, DC 20060, USA; E-Mail: tobesisan@howard.edu; 2 W. Montague Cobb, NMA Institute, 1012 Tenth St. NW, Washington, DC 20001, USA; E-Mail: njarrett@nmanet.org

**Keywords:** access to care, prevention, hispanics, blacks

## Abstract

In order to elucidate cultural correlates of utilization of primary health services by young adult men, we investigated religion in which one was raised and service utilization. Using data from a national survey we tested the hypothesis that religion raised predicts access to and utilization of a regular medical care provider, examinations, HIV and other STD testing and counseling at ages 18–44 years in men born between 1958 and 1984. We also hypothesized that religion raised would be more predictive of utilization for Hispanic Americans and non-Hispanic Black Americans than for non-Hispanic White Americans. The study included a national sample of 4276 men aged 18–44 years. Descriptive and multivariate statistics were used to assess the hypotheses using data on religion raised and responses to 14 items assessing health care access and utilization. Compared to those raised in no religion, those raised mainline Protestant were more likely (p < 0.01) to report a usual source of care (67% *vs.* 79%), health insurance coverage (66% *vs.* 80%) and physical examination (43% *vs.* 48%). Religion raised was not associated with testicular exams, STD counseling or HIV testing. In multivariate analyses controlling for confounders, significant associations of religion raised with insurance coverage, a physician as usual source of care and physical examination remained which varied by race/ethnicity. In conclusion, although religion is a core aspect of culture that deserves further study as a possible determinant of health care utilization, we were not able to document any consistent pattern of significant association even in a population with high rates of religious participation.

## 1. Introduction

The United States continues to experience longstanding, major disparities in health status and utilization of health services [1]. With the introduction of nearly universal health insurance for persons aged 65 and above more than four decades ago, the nation took a major first step towards addressing its socioeconomic, racial and ethnic health disparities. However, progress has been slow towards taking the next steps and providing insurance for persons under age 65, even as rapid advances in ever more expensive medical technology create, maintain or widen disparities [2]. Although the national goal of reducing or eliminating disparities cannot be achieved without that next step, a large literature has grown up addressing various aspects of disparities and their putative causes. Non-institutionalized young men are especially likely to lack health insurance and have limited access to primary care [3]. Cultural factors are receiving more attention as barriers to access to and utilization of quality health services by minorities and programs are being developed and evaluated to reach underserved groups [3–5]. However, little attention has been given by researchers to religion, a core aspect of culture, despite its pervasive and well-documented effects on health behaviors and outcomes, especially among Black Americans [6–8].

A number of studies suggest that utilization of preventive services is associated with greater religious participation [9–14]. A recent study indicated that religious practice was associated with having a usual source of care [15]. Other studies found a strong positive relationship of religiousness with trust in one’s physician [16,17]. Some have postulated that religious training equips young persons with life skills such as discipline, persistence, and obedience that facilitate health preserving behaviors such as utilization of preventive care [15]. A few congregations actually provide health services. Members may get support and information from other members including health professionals which promotes use of services [12,18]. Most previous research is limited to adults over 50 years of age and/or women’s health issues.

In order to further elucidate the role of religion as a cultural correlate of appropriate health service utilization, we investigated religion and utilization of primary health services by young adult men, for whom little or no information is available. Using data from a national survey we tested the hypothesis that the religion in which one was raised predicts having a usual source of care, having an office-based physician as usual source of care, having health insurance coverage, and having physical examinations, HIV and other STD testing and counseling 10–20 years later in men aged 18–44 born between 1958 and 1984.

We used data from the National Survey of Family Growth Cylce 6 conducted in March, 2002-February, 2003 on a nationwide multi-stage probability sample of men in the household population aged 15–44 years of the United States yielding 4,928 interviews with a response rate of 79%. Teens, young adults (20–24 years), and black and Hispanic persons were oversampled. Details of the survey plan, sample design, operations, response rates, imputation of missing data, Institutional Review Board approval, and public release of data have been published as have procedures used to obtain informed consent and to maintain confidentiality of information [19].

Demographic data including self-reported race category and Hispanic ethnicity, years of education completed, and beliefs and attitudes about family were collected by household interview using computed-assisted personal interviews administered by interviewers [19]. Men were asked, “Now I have a few questions about religion. In what religion were you raised, if any? (None, Catholic, Jewish, Southern Baptist, Methodist, African Methodist, Lutheran, Presbyterian, Episcopal, LDS/Mormon and Other). Categories were collapsed to form analysis categories None, Catholic, Baptist, Other Protestant, and Other for analyses of the religion in which they were raised, if any, and change between religion raised and their current affiliation, if any. We excluded 633 men aged 15–17, who might still be living at home under parental influence in both religious and health care matters. We analyzed data on the 4,275 of 4,295 men 18–44 years of age who had complete data on religion raised and having a usual source of health care.

Detailed weighted descriptive statistics were computed. All analyses were performed using the SUDAAN software package, with techniques that incorporated sampling weights and design features of the survey [20]. PROC RLOGIST was used for logistic regression analysis. A hierarchical modeling strategy was pursued with confounding variables chosen based on literature review.

## 2. Results and Discussion

At ages 18–44, 8% of men were raised in no religion, 36% were raised Catholic, 32% were raised Baptist, 17% were raised other Protestant, and 7% were raised in a non-Christian religion. Table 1 shows the distribution of health care access and utilization variables across groups of religion raised. Compared to those raised in no religion, those raised other Protestant were more likely (p < 0.01) to report a usual source of care (67% *vs.* 79%), uninterrupted health insurance coverage during the past year (66% *vs.* 80%) and physical examination (43% *vs.* 48%). Religion raised was not significantly associated with testicular exams, STD counseling or HIV testing.

Table 2 shows the results of logistic regression analyses with uninterrupted insurance coverage in the past 12 months (yes/no) as outcome variable and religion raised, age, and race/ethnicity as independent variables. In a model with religion raised, age and race/ethnicity and all interactions, a chunk test revealed a least one significant interaction (p < 0.03). Further analysis revealed borderline significant interactions of age with race/ethnicity and race/ethnicity with religion raised. Guided by stratified analyses above, a model controlling for age was fit for non-Hispanic whites and also for Hispanics and non-Hispanic blacks combined. Controlling for age, religion raised remained a significant predictor of having a coverage in non-Hispanic whites (p = 0.0001).

Compared to those raised in no religion, those raised other Protestant had an odds ratio of 1.75 (95% CL 1.11–2.77, p = 0.02). In Hispanics and blacks, religion raised was also a significant predictor of coverage (p = 0.0006). However the pattern of main effects was different (Table 2). Those raised Baptists and other Protestants were both more likely to be covered than those raised in no religion, whereas in non-Hispanic whites those raised Baptist tended to be less likely than those raised in no religion. After controlling for marital status and education in addition to age, religion raised remained a significant predictor of coverage for non-Hispanic whites (p = 0.0001) and for Hispanics and non-Hispanic blacks (p = 0.0016). However also controlling for Hispanic ethnicity rendered the association non-significant in the latter group (p = 0.70) (Table 2).

Logistic regression analyses with usual source of care (yes/no) as outcome variable and religion raised, age, and race/ethnicity as independent variables was performed. In a model with religion raised, age and race/ethnicity and all interactions, a chunk test revealed a least one significant interaction (p < 0.01). Further analysis revealed a significant interaction of age and race/ethnicity, but no significant interaction of age or race/ethnicity with religion raised. Therefore all models for usual source of care included age and age × race/ethnicity. Controlling for age, race/ethnicity, and age × race/ethnicity, religion raised was no longer a significant predictor of having a usual source of care (p = 0.28). Compared to those raised in no religion, those raised other Protestant had an odds ratio of 1.66 (95% CL 0.97–2.82, p = 0.06).

Among those with a usual source of care, 59% reported having an office-based physician as the source of care. For the outcome of having a regular physician, bivariate analyses showed higher odds for those raised Catholic, Baptist and Other Protestant compared to no religion (p < 0.01). However, there was an interaction of religion with race/ethnicity. In blacks and Hispanics combined after controlling for age and ethnicity, this association was significant (p = 0.007) with those raised Baptists and other Protestants having higher odds than those raised in no religion. After controlling in addition for marital status and education, significance was maintained (p = 0.02) for Baptists and for other Protestants (Table 3). After further adjustment for insurance coverage, results were essentially the same (Baptists OR = 1.78, 0.86–3.66; other Protestants OR = 3.39, 1.48–7.79). In non-Hispanic whites after adjusting for age or age, marital status and education, religion raised was significantly associated with having a regular physician (Table 3). After further adjustment for insurance coverage, those raised Catholic (OR = 2.06, 1.22–3.46), Baptist (OR = 1.70, 1.09–2.66) and other Protestant (OR = 1.65, 1.01–2.68) were still more likely to have a regular physician at interview.

Logistic regression analyses with history of having a physical examination (yes/no) as outcome variable and religion raised, age, and race/ethnicity as independent variables was performed. In a model with religion raised, age and race/ethnicity and all interactions, a chunk test revealed a least one significant interaction (p < 0.006). Further analysis revealed borderline significant interactions of race/ethnicity with religion raised (p = 0.06). Guided by stratified analyses above, a model controlling for age was fit for non-Hispanic whites and also for Hispanics and non-Hispanic blacks combined. Controlling for age, religion raised remained a significant predictor of having an exam in non-Hispanic whites (p = 0.007). Catholics were least likely to have had a physical examination (Table 4). In Hispanics and blacks, religion raised was also a significant predictor (p < 0.0001). However the pattern of main effects was different (Table 4). After controlling for marital status, education and Hispanic ethnicity in addition to age, religion raised remained a significant predictor of examination for non-Hispanic whites (p = 0.006) and for Hispanics and non-Hispanic blacks (p < 0.004) (Table 4). Those raised Catholic, Baptist and other Protestant were less likely to have had an examination than those raised in no religion. After also controlling for insurance coverage, religion raised maintained its significance for non-Hispanic whites (p = 0.02) and for Hispanics and non-Hispanic blacks (p = 0.02).

To assess the effect of changing religiousness between adolescence and adulthood, a variable with the following categories was created: (I) not raised in any religion and no religious affiliation at interview (5.5%), (II) not raised in any religion but having a religious affiliation at interview or raised in a religion but not having a religion affiliation at interview (15.6%), and (III) raised in a religion and having a religious affiliation at interview (78.9%). In bivariate analyses, those in group III were most likely to report having uninterrupted insurance coverage (p = 0.004) and physical exam (0.04). In those aged 18–44, men in group II (OR 0.61, 0.49–0.76) but not I (OR 0.84, 95% CL 0.57–1.22) were less likely to have uninterrupted health insurance coverage compared to those in group III after adjusting for age and race/ethnicity (p = 0.0002). After controlling for education and marital status in addition to age and race/ethnicity left OR and CL essentially unchanged. In similarly adjusted analyses, having had a physical examination showed no significant association with change in affiliation (p = 0.08). Thus men who changed affiliation were less likely to have uninterrupted coverage than those who did not.

In bivariate analyses, those in group III were most likely to report having a usual source of care (p = 0.004), and a regular office-based physician (0.001). In Hispanics and non-Hispanic blacks, group I was least likely to have a regular source of care, physician or uninterrupted coverage or examination, but in non-Hispanic whites group II was least likely. However, in logistic regression, the interaction of race/ethnicity with religious change was not significant, perhaps because numbers of Hispanics and blacks in category I was quite small (<3%). A significant interaction with age was seen (p < 0.01). Therefore further analyses were done within age groups.

In men aged 18–34, men in groups I (OR 0.55, 95% CL 0.33–0.94) and II (OR 0.62, 0.46–0.84) were less likely to have a usual source of care compared to those in group III after adjusting for age and race/ethnicity. After controlling for education and marital status in addition to age and race/ethnicity left OR and CL essentially unchanged. Findings for having an office-based physician as usual source of care were similar: adjusted prevalence and OR (CL) were group I 26%, OR = 0.49 (0.29–0.82), group II 33%, OR = 0.67 (0.48–0.94) and group III 42%, OR = 1.00, remaining significant even after also controlling for insurance coverage (p = 0.01). At ages 35–44, a similar pattern was of borderline significance after controlling for all confounders (p = 0.08). Hence both being raised in a religion and remaining in a religion were important predictors of having a usual source of care and of having an office-based physician as usual source of care.

### 2.1. Discussion

These results provide little evidence in support of our hypothesis that in men aged 18–45, religion raised is predictive of future acquisition of uninterrupted health insurance coverage and any usual source of care and an office-based physician as usual source of care, and physical examination in the year before interview. An independent association of religion raised with insurance coverage was found, but the pattern varied by race and ethnicity. The stronger effect of being raised Baptist or other Protestant among Hispanics and non-Hispanic blacks seems consistent with well-known patterns of greater religiousness in black and Hispanic Protestants compared to non-Hispanic whites. The association with usual source of care was not independent of age and race/ethnicity. However, an independent association with having a regular office-based physician was found. However the results for having a physical examination and other services were not consistent with these findings and indicated that men raised in a religion might be similarly likely to utilize some primary services such as physical examination compared to those raised in a religion. Analysis of changes in religious affiliation between adolescence and adulthood were consistent with a positive association of ongoing affiliation and access to care. In sum, although religion is a core aspect of culture that may deserve further study as a possible determinant of health care utilization, we were not able to document in this secondary data analysis any consistent pattern of significant association even in a population with fairly high rates of religious participation.

Prior studies have not considered young men and present results are not directly comparable to those for older persons and women [9–15]. Limitations of the present study include possible bias arising from survey non-response and from missing values for some variables. Attempts to minimize such bias included using trained interviewers, interviewing over a third of Hispanic respondents in Spanish, and using computerized personal interviewing and audio-assisted, computerized self interviewing, which resulted in low frequencies of imputed values due to missing data, e.g., only 7.6% for poverty index, about half that seen in earlier survey cycles. Possible information bias may result from use of self reported data. For example, reflecting the “social desirability effect,” modest over-reporting of religious rearing is possible as has been shown for attendance [21]; however with systematic over-reporting unrelated to outcome variables, bias should be towards the null. Bias due to confounding by variables not controlled for cannot be excluded. Nor could several possible unmeasured mediating factors be assessed. For example, data were lacking in this survey on health status, functional status and social support.

Despite the large overall sample size in this survey, reliability of estimates and statistical power was limited for some subgroups (e.g., Black Catholics and Hispanics with no religion). Further, the large sample may contribute to the appearance of significant results that have no practical importance. The representativeness of the sample and the use of sample weights provides generalizability of the results to United States household population of the same ages. Although this was a cross-sectional survey, temporal sequence was assessed by using religion in which one was raised to predict later service utilization in the 12 months prior to interview.

## 3. Conclusions

In a large national sample, the few statistically significant findings (e.g., persons raised in mainline Protestant religion were independently more likely to report a usual source of care, health insurance coverage, and physical examination compared to those raised in no religion among American men born in 1958–1985) followed no consistent pattern and may have been due to chance or large sample size rather than reflecting important associations of a causal nature. Utilization of preventive services studied were not associated with religion raised.

## Figures and Tables

**Table 1.** Health care access, utilization and selected other variables (percents) by religion raised in men aged 18–44 in 2002.

Variable		Mean					
		N	None	Catholic	Baptist	Other Protestant	Other
Total		4,275	325	1,623	1,429	610	288
Age	18–24	1,419	11	36	34	13	7
	25–34	1,428	7	36	33	16	8
	35–44	1,428	7	36	30	20	7
Ethnic group*	HA	988	3	81	12	2	2
	WA	2,232	9	30	32	22	6
	BA	812	5	10	60	12	5
	Other	243	9	20	28	6	36
Education	≤HS	2,227	7	39	36	13	5
	>HS	2,048	9	33	29	20	10
Marital status*	Married	1,227	6	35	34	18	7
	Not married	3,048	9	37	31	16	7
Usual source	Yes	2,932	67	68	72	79	73
care	No	1,343	33	32	28	21	27
Insured all last 12	Yes	1,419	66	66	67	80	66
months	No	2,848	34	34	33	20	33
Physical exam	Yes	1,996	43	48	44	48	36
	No	2,779	57	52	56	52	64
Testicular exam	Yes	1,623	36	38	35	40	30
	No	2,650	64	62	65	60	70
HIV test	Yes	2,089	49	50	46	47	51
	No	2,155	50	50	46	52	47
STD counseling	Yes	569	7	11	10	8	8
	No	3,705	93	89	90	92	92

* n × 2 Chi-square p < 0.01; + P < 0.05; WA, non-Hispanic White American, BA, non-Hispanic Black American, HA, Hispanic American.

**Table 2.** Logistic regression analysis (adjusted prevalence, odds ratio and 95% confidence limits) of religion raised as a predictor of uninterrupted health insurance coverage in the past 12 months.

		**Age-adjusted prevalence (%)**	**Age-adjusted odds ratio**	Adjusted* odds ratio
Non–Hispanic white	Religion raised			
N = 2,227	None	72	1.00	1.00
	Catholic	75	1.15 (0.75–1.76)	1.21 (0.78–1.87)
	Baptist	68	0.82 (0.53–1.25)	0.81 (0.52–1.26)
	Other Protestant	82	1.75 (1.11–2.77)	1.81 (1.12–2.92)
	Other	65	0.72 (0.38–1.38)	0.73 (0.37–1.41)
Hispanic and non–Hispanic black n =1,904				
	None	54	1.00	1.00
	Catholic	54	1.03 (0.54–1.95)	1.28 (0.66–2.51)
	Baptist	68	1.81 (0.98–3.36)	1.31 (0.70–2.47)
	Other Protestant	72	2.17 (0.94–5.03)	1.41 (0.60–3.30)
	Other	57	1.12 (0.44–2.86)	0.77 (0.29–2.09)

* adjusted for age, education, marital status (and Hispanic ethnicity in the Hispanic/black group).

**Table 3.** Logistic regression analysis (adjusted prevalence, odds ratio and 95% confidence limits) of religion raised as a predictor of having an office-based physician as usual source of care in the past 12 months in men aged 18–44 years.

		**Age–adjusted prevalence (%)**	**Age–adjusted odds ratio**	Adjusted* odds ratio
Non–Hispanic white	Religion raised			
N = 2,232	None	35	1.00	1.00
	Catholic	53	1.98 (1.22–3.21)	2.08 (1.24–3.48)
	Baptist	47	1.60 (1.04–2.48)	1.64 (1.05–2.57)
	Other Protestant	49	1.74 (1.10–2.75)	1.77 (1.08–2.90)
	Other	47	1.66 (0.95–2.92)	1.72 (0.98–3.00)
Hispanic and non–Hispanic black n = 1,913				
	None	22	1.00	1.00
	Catholic	27	1.57 (0.74–3.32)	1.40 (0.67–2.94)
	Baptist	36	2.03 (0.97–4.26)	1.82 (0.88–3.79)
	Other Protestant	50	4.02 (1.76–9.16)	3.31 (1.46–7.50)
	Other	29	1.67 (0.61–4.58)	1.35 (0.52–3.54)

* adjusted for age, education, marital status, insurance coverage (and Hispanic ethnicity in the Hispanic/black group).

**Table 4.** Add a descriptive label of the table here Logistic regression analysis (adjusted prevalence, odds ratio and 95% confidence limits) of religion raised as a predictor of having a physical examination in the past 12 months.

		**Age-adjusted prevalence (%)**	**Age-adjusted odds ratio**	Adjusted* odds ratio
Non–Hispanic white n = 2,231	Religion raised			
	None	60	1.00	1.00
	Catholic	49	0.63 (0.39–1.03)	0.62 (0.38–0.99)
	Baptist	63	1.11 (0.66–1.87)	1.09 (0.65–1.82)
	Other Protestant	54	0.76 (0.45–1.31)	0.76 (0.44–1.31)
	Other	61	1.05 (0.53–2.06)	1.05 (0.55–2.02)
Hispanic and non–Hispanic black n = 1,913				
	None	53	1.00	1.00
	Catholic	54	1.03 (0.51–2.07)	0.78 (0.38–1.64
	Baptist	41	0.61 (0.32–1.16)	0.82 (0.40–1.66)
	Other Protestant	36	0.48 (0.21–1.10)	0.71 (0.28–1.77)
	Other	66	1.69 (0.63–4.58)	2.38 (0.86–6.58)

* adjusted for age, education, marital status (and Hispanic ethnicity for Hispanic/black group).
